# A potential kidney - bone axis involved in the rapid minute-to-minute regulation of plasma Ca^2+^

**DOI:** 10.1186/s12882-015-0019-3

**Published:** 2015-03-15

**Authors:** Anders Nordholm, Maria L Mace, Eva Gravesen, Klaus Olgaard, Ewa Lewin

**Affiliations:** Nephrological Department B, Herlev Hospital, DK 2730 Copenhagen, Denmark; Nephrological Department P, Rigshospitalet, University of Copenhagen, Copenhagen, Denmark

**Keywords:** Plasma Ca^2+^ regulation, Minute-to-minute regulation, Kidney, Bone, Kidney-Bone-Axis, FGF23, Acute uremia

## Abstract

**Background:**

Understanding the regulation of mineral homeostasis and function of the skeleton as buffer for Calcium and Phosphate has regained new interest with introduction of the syndrome “Chronic Kidney Disease-Mineral and Bone Disorder”(CKD-MBD). The very rapid minute-to-minute regulation of plasma-Ca^2+^ (p-Ca^2+^) takes place via an exchange mechanism of Ca^2+^ between plasma and bone. A labile Ca storage pool exists on bone surfaces storing excess or supplying Ca when blood Ca is lowered. Aim was to examine minute-to-minute regulation of p-Ca^2+^ in the very early phase of acute uremia, as induced by total bilateral nephrectomy and to study the effect of absence of kidneys on the rapid recovery of p-Ca^2+^ from a brief induction of acute hypocalcemia.

**Methods:**

The rapid regulation of p-Ca^2+^ was examined in sham-operated rats, acute nephrectomized rats(NX), acute thyroparathyrectomized(TPTX) rats and NX-TPTX rats.

**Results:**

The results clearly showed that p-Ca^2*+*^ falls rapidly and significantly very early after acute NX, from 1.23 ± 0.02 to 1.06 ± 0.04 mM (p < 0.001). Further hypocalcemia was induced by a 30 min iv infusion of EGTA. Control groups had saline. After discontinuing EGTA a rapid increase in p-Ca^2+^ took place, but with a lower level in NX rats (p < 0.05). NX-TPTX model excluded potential effect of accumulation of Calcitonin and C-terminal PTH, both having potential hypocalcemic actions. Acute TPTX resulted in hypercalcemia, 1.44 ± 0.02 mM and less in NX-TPTX rats,1.41 ± 0.02 mM (p < 0.05). Recovery of p-Ca^2+^ from hypocalcemia resulted in lower levels in NX-TPTX than in TPTX rats, 1.20 ± 0.02 vs.1.30 ± 0.02 (p < 0.05) demonstrating that absence of kidneys significantly affected the rapid regulation of p-Ca^2+^ independent of PTH, C-PTH and CT.

**Conclusions:**

P-Ca^2+^ on a minute-to-minute basis is influenced by presence of kidneys. Hypocalcemia developed rapidly in acute uremia. Levels of p-Ca^2+^, obtained during recovery from hypocalcemia resulted in lower levels in acutely nephrectomized rats. This indicates that kidneys are of significant importance for the ‘set-point’ of p-Ca^2+^ on bone surface, independently of PTH and calcitonin. Our results point toward existence of an as yet unknown factor/mechanism, which mediates the axis between kidney and bone, and which is involved in the very rapid regulation of p-Ca^2+^.

## Background

Calcium (Ca) has important extracellular as well as intracellular functions. Its extracellular functions include its role in blood clotting, maintenance of plasma membrane integrity and intercellular adhesion. Extracellular Ca provides a source of calcium ions (Ca^2+^) essential for intracellular processes. Furthermore, extracellular Ca^2+^ participates in its own regulation through systemic and local Ca^2+^ sensing receptor (CaR) mediated actions in tissues related to the mineral ion homeostasis [1,2]. Finally, extracellular Ca provides a constant supply to the exchange of Ca within the large reservoir of Ca - the skeleton. The skeleton represents the largest compartment of total body Ca, containing more than 99%.

The free plasma Ca^2+^ concentration is in normal mammals kept within a very narrow range in the circulating blood, except for small diurnal variations [2-5]. The detailed regulation of plasma Ca^2+^ on the minute-to-minute basis is, however, still only incompletely understood. Plasma Ca homeostasis is regulated through a complex interplay between different calciotropic hormones, where deviations in plasma Ca^2+^ concentration are sensed by CaR [2]. The CaR then regulates plasma Ca^2+^ concentration through direct or indirect actions on the calcium translocating tissues, bone, intestine and kidney and via regulation of PTH, calcitriol, and calcitonin (CT) [2].

The traditional understanding of the Ca homeostasis provides the following mechanism for the subsequent normalization of plasma Ca^2+^ after a brief induction of hypocalcemia [6-8]. Even a slight reduction in the extracellular Ca^2+^ concentration elicits a prompt increase in the rate of PTH secretion. The renal responses to the increased plasma PTH level are phosphaturia, enhanced distal tubular reabsorption of Ca^2+^ and increased generation of the active metabolite of vitamin D, 1,25(OH)_2_D (calcitriol), from 25(OH)D by stimulation of the 1α-hydroxylase in the proximal convoluted tubules. The increased level of calcitriol stimulates the intestinal absorption of phosphate (P) and Ca. PTH and calcitriol promote net release of P and Ca from bone. The increased flux of Ca^2+^ into the extracellular fluid, coupled with renal retention of Ca^2+^ restore circulating Ca^2+^ levels toward normal, and thereby inhibits PTH secretion and close the negative feedback loop. In this model of Ca^2+^ homeostasis the emphasis is on the key role of PTH and kidney. This classical model of plasma Ca^2+^ regulation does indeed apply to the long-term regulation and stability of plasma Ca^2+^, but does not apply to the very rapid minute-to-minute regulation of plasma Ca^2+^, as previously clearly shown from our lab. [9-12].

The conceptual framework on how plasma Ca^2+^ is held constant has been expanded by Kurokawa [13], who described an elegant concept, which combined the set point for the Ca^2+^ flux between extracellular fluid volume (ECF) and bone, and the set point for renal tubular Ca^2+^ reabsorption in relation to the set point for the Ca^2+^ regulated PTH secretion from the parathyroids [13]. The interplay between PTH and calcitriol will effectively set the Ca^2+^ level in ECF by adjusting the set points in the different organs to the same level of Ca^2+^. As such the set points of the Ca^2+^ regulating tissues correspond to the resulting plasma Ca^2+^ level, which the organism is maintaining. This elegant concept is strongly supported by results from studies on the regulation of renal tubular reabsorption of Ca^2+^ [7,14,15]. The site along the nephron where the set point for calcium reabsorption is present and regulated appears to be in the distal nephron segments, where the PTH/PTHrP receptor (PTH1R) and the vitamin D receptor (VDR) are located [16-18] together with the vitamin D inducible calcium-binding protein, calbindin-D28K, Klotho and the epithelial Ca^2+^ channel, TRPV5 [19,20].

Recently, a new factor in the rapid regulation of Ca^2+^ homeostasis has been proposed, as fibroblast growth factor 23 (FGF23) was shown to promote renal Ca^2+^ reabsorption through the TRPV5 channel in the distal tubule, and hereby contributes to conserve Ca. In short time *in vitro* experiments FGF23 increased the TRPV5 protein abundance and the channel activity already after 45 minutes [21].

How and where the calciotropic hormones are setting the set point for Ca^2+^ between bone and the ECF interphase is less clear. Kurokawa assumed that PTH and calcitriol, in concert, adjusted the set point for Ca^2+^ by regulating the rate of the coupled bone resorption and formation [13]. However, the amount of calcium conserved by distal tubules is minimal and the regulation of the rate of bone turnover time consuming; as such these mechanisms can’t be representative for the main mechanism involved in the rapid minute-to-minute regulation of plasma Ca^2+^.

The existence of a labile pool of Ca^2+^ at the quiescent surface of bone which is in dynamic equilibrium with ECF has previously clearly been established by experiments using incorporation of radiolabelled Ca in bone. The magnitude of the rapid calcium exchange was estimated to be many fold higher than the daily flux from remodeling based bone turnover. This labile pool functions as a short-term buffer, taking up or releasing calcium to correct for changes in ECF [22]. Knowledge concerning this process, as summarized in details by Parfitt, has with some exceptions [2,9,10,12,22-25], disappeared from the current texts [22,26].

Results from our lab have previously clearly shown that neither PTH, calcitriol or CT were essential for the rapid minute-to-minute regulation of plasma Ca^2+^ [9-12]. In these studies the rapid recovery of plasma Ca^2+^ was examined after a brief induction of hypocalcemia with an EGTA infusion in parathyroidectomized (PTX), thyroidectomized (TX), thyroparathyroidectomized (TPTX) and calcitriol depleted rats and rats treated with different doses of PTH, CT and calcitriol. The overall conclusion was that none of these calciotropic hormones were prerequisite for the rapid correction of plasma Ca^2+^ [9-12]. Our results did, however as expected, clearly show a significant impact of these calciotropic hormones on the long-term plasma Ca^2+^ homeostasis, with the calciotropic hormones defining the plasma Ca^2+^ levels, by adjusting the set point for plasma Ca^2+^ in a dose-related manner.

Furthermore, we have previously shown that the rapid calcemic recovery, which occurs after a brief induction of hypocalcemia does not result from suppression of CT levels. Thus, the calcemic recovery curves were similar in TPTX rats, normal and PTX rats and in rats given an exogenous salmon CT infusion. It was also clearly shown that TPTX and selective TX resulted in similar increments of plasma Ca^2+^. This indicated that the effect of removing endogenous CT initially dominated over the effect of removing endogenous PTH, and resulted in an increase of calcium efflux from bone to the extracellular fluid. The role of PTH in the rapid, minute-to-minute plasma calcium regulation was characterized by stable plasma Ca^2+^ for 2 h after acute PTX [10]. It was also shown, that presence or absence of CT had no influence on the rapid calcemic recovery. However, an immediate severe hypercalcemia, that occurred after deprivation of CT, indicated that CT had an acute effect on plasma Ca^2+^ levels, and as such that calcium homeostasis was hormone-controlled, even on a minute-to-minute basis [25]. Thus, our previous results clearly pointed towards that the involvement of an exchangeable calcium pool on the surface of bone was under hormonal control and not just representing a physical - chemical equilibrium between calcium crystals and extracellular fluid, and in this way was keeping the extracellular calcium concentration very stable [22].

The understanding of the regulation of the mineral homeostasis and the function of the skeleton as a buffer for Ca and P has regained new interest with the introduction of the concept of the syndrome “Chronic Kidney Disease - Mineral and Bone Disorder” (CKD-MBD) by Moe et al. [27]. This concept emphasizes the important relationship between disturbed mineral homeostasis in uremia and diminished ability of bone to accumulate Ca and P, and it’s relation to the development of severe complications, mainly vascular calcifications, that are so characteristic for the uremic condition and which are related to a substantial risk of increased morbidity and mortality [28].

Hypocalcemia is an important complication in uremia. Traditionally, hypocalcemia in uremia is explained by the associated decreased renal biosynthesis of calcitriol, P retention, decreased intestinal Ca absorption, and skeletal resistance to PTH, factors that all take time to respond. However, a very rapid skeletal response to the induction of acute kidney injury has recently been demonstrated [29], where an acute kidney injury within one hour resulted in a significant increase in the skeletal derived phosphaturic hormone, FGF23 [29]. The timing of this elevation of FGF23 might indicate the existence of a kidney-bone axis, which is responding very rapidly to kidney injury. Previously a parathyroid – bone axis has been shown where PTX lead to a rapid decrease in serum FGF23 and where exogeneous PTH within hours lead to an increase in FGF23 [30], expanding the understanding of a close relation between bone and important hormones in the calcium homeostasis.

Thus, the present investigation was conducted in order to examine the performance of plasma Ca^2+^ in the very early phase of acute uremia, as induced by total bilateral nephrectomy and to study the effect of total absence of kidneys on the rapid recovery of plasma Ca^2+^ from a brief induction of acute hypocalcemia.

## Methods

### Animals

The experimental studies were performed in accordance with the National Institute for Health Guide for Care and Use of Laboratory Animals and approved by the Animal Experiments Inspectorate, the Ministry of Food, Agriculture and Fisheries, Denmark (Reference: 2012-DY-2934-00023). Adult male Wistar and DA rats weighing 250 g (Taconic A/S, Ejby, Denmark) were housed in a temperature-controlled environment with a 12-hour light/dark circle. The rats were given ad libitum access to a standard diet (0.9% calcium, 0.7% phosphorus and 600 IU vitamin D per kg diet; Altromin, Spezialfutter GmbH & Co., KG, Germany) and water. Rats were anesthetized with pentobarbital (50 microg/kg intraperitoneally; Nycomed-DAK, Copenhagen, Denmark). Additional doses were administered when required in order to maintain a steady level of anesthesia. Since DA and Wistar rats had identical response of Ca^2+^ to nephrectomy and recovery from acute hypocalcemia, the data are presented combined. The rats were not fasting before the experiments.

### Thyroparathyroidectomy (TPTX)

Acute TPTX was performed under stereomicroscope using a microsurgical procedure with special care to avoid trauma to the recurrent laryngeal nerve. The model has previously been described from our lab [12]. Successful TPTX was confirmed by no detectable circulating PTH and CT one hour after the operation [12].

### Bilateral nephrectomy (NX)

NX was performed from the back by a one-step surgical removal of both kidneys [31], as previously described [9]. The sham procedure consisted of incision in the skin only.

### Induction of hypocalcemia by an EGTA infusion and plasma measurements

Hypocalcemia was induced by an intravenous infusion of EGTA (ethylene-bis(oxyethylenenitrilo)tetraacetic acid; Sigma, USA), 40 mM, at a rate of 3.0 ml/h for 30 minutes (from time 60 to 90 minutes) through a catheter inserted into the femoral vein. Plasma Ca^2+^ was measured by a Calcium Selective Electrode (ABL505, Radiometer, Copenhagen, Denmark). Plasma phosphate was analyzed by Vitros 250 (Ortho-Clinical Diagnostics, Raritan, NJ, USA).

### Design

Rats were allocated to eight experimental models, and the rapid regulation of plasma Ca^2+^ was examined in sham-operated rats, NX rats, TPTX rats and NX-TPTX rats, as presented in Table 1. The NX-TPTX model was introduced in order to exclude the potential effect of accumulation of CT and C-terminal PTH fragments, both having potential hypocalcemic actions on the acute uremia associated hypocalcemia [32,33]. The control groups were given infusion of saline instead of EGTA. The rats were sham operated, NX, TPTX or NX-TPTX at time 0, just after obtaining the time 0 blood samples. A short lasting infusion of EGTA or saline was given via the femoral vein from time 60 to 90 minutes. Samples for determination of plasma Ca^2+^ and phosphate concentrations were obtained from a catheter in the femoral artery at times 0, 60, 90, 100, 120, 140 min. After each blood sample, the blood volume was replaced by infusion of 0.4 ml NaCl. The hypocalcemic model is described in more details from our lab [10].

**Table 1:** **Rats were divided into four groups each subdivided into receiving either NaCl or EGTA**

	**Sham**		**NX**		**TPTX**		**NX-TPTX**	
	*NaCl*	*EGTA*	*NaCl*	*EGTA*	*NaCl*	*EGTA*	*NaCl*	*EGTA*
*n:*	10	8	7	10	18	16	10	10

Number of rats (n) enrolled is specified.

### Statistical analysis

The data are presented as mean ± SEM and compared with Mann–Whitney test or t-test,. *P* < 0.05 is considered statistically significant.

## Results

**Figure 1:**
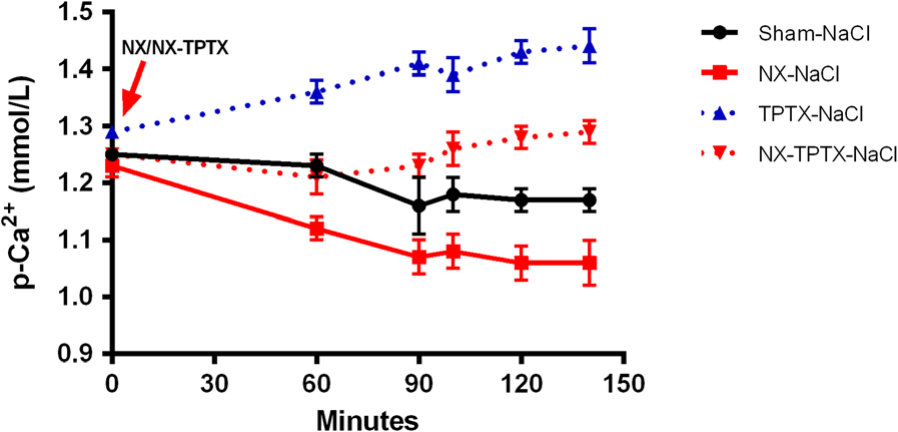
**Rapid development of hypocalcemia in total nephrectomized (NX) rats.** Plasma Ca^2+^ concentrations in sham, NX, TPTX, and NX-TPTX rats. Plasma Ca^2+^ in the NX rats declined rapidly with development of significant hypocalcemia already within 60 minutes, as compared to sham rats (p < 0.01). TPTX resulted as expected in significant hypercalcemia (p < 0.05) within 60 minutes. The increase in plasma Ca^2+^ was significantly lower in NX-TPTX rats, than in TPTX rats, (p <0.05).

The effect of NX on plasma Ca^2+^ concentrations is illustrated in Figure 1. NX resulted in a rapid development of significant hypocalcemia. Already at 60 minutes plasma Ca^2+^ was significantly diminished in NX rats compared to sham rats (p < 0.01). Then a further reduction of plasma Ca^2+^ took place. Plasma Ca^2+^ diminished in NX rats from a basal value of 1.23 ± 0.02 to 1.06 ± 0.04 mmol/L at 140 min, p < 0.001, while sham rats, given saline only, had a slight decrease in plasma Ca^2+^ from 1.21 ± 0.01 to 1.17 ± 0.02 mmol/L (ns). TPTX resulted as expected in hypercalcemia with a significant increase in plasma Ca^2+^ from 1.29 ± 0.01 to 1.44 ± 0.02 mmol/L (p < 0.001). The increase in plasma Ca^2+^ was significantly less in NX-TPTX rats, 1.29 ± 0.02 mmol/L, than in TPTX rats, (p < 0.05) (Figure 1).

Further the minute-to-minute regulation of plasma Ca^2+^ was examined after the EGTA induced hypocalcemia. In sham rats significant hypocalcemia with plasma Ca^2+^ falling from 1.21 ± 0.01 to 0.81 ± 0.03 mmol/l (p < 0.001) developed within 30 minutes of EGTA infusion. Then subsequently a rapid and significant increase of plasma Ca^2+^ took place within 10 minutes after stopping the EGTA infusion and with further recovery towards basal levels.

**Figure 2:**
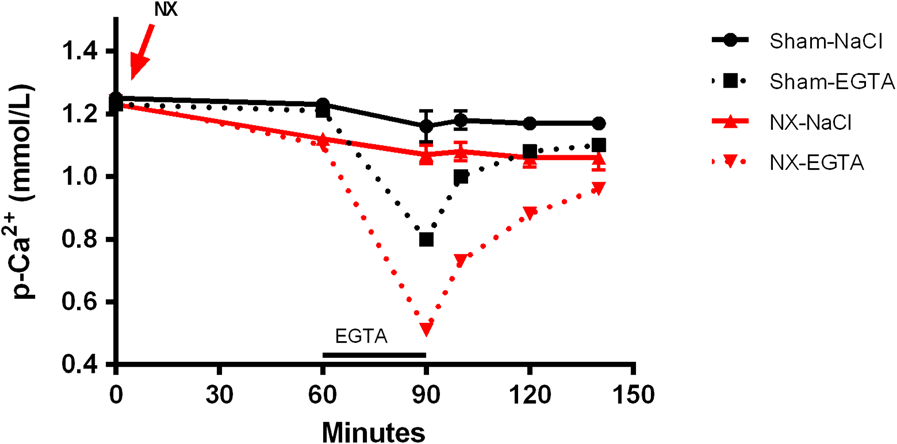
**Impact of the kidney on recovery of plasma Ca** ^**2+**^ **from acute hypocalcemia.** NX rats had a significant lower nadir of EGTA induced hypocalcemia than EGTA infused sham rats (p < 0.01). During the recovery from hypocalcemia the concentration of plasma Ca^2+^ remained at all time points significantly lower in the NX group compared to the sham group (p < 0.01).

In order to examine any effect of the presence of kidneys on the rapid minute-to-minute plasma Ca^2+^ regulation, sham operated rats were compared to total bilateral NX rats, as shown in Figure 2. The EGTA infused NX rats had a significant lower nadir of hypocalcemia, as compared to sham rats, 0.51 ± 0.02 vs. 0.80 ± 0.02 mmol/L, (*p* < 0.01) and the concentration of plasma Ca^2+^ remained significantly lower in the NX group during the recovery from hypocalcemia, (*p* <0.01) at time 100, 120 and 140 minutes. Thus in the presence of acute alterations in plasma Ca^2+^ concentration, NX rats had the ability to restore calcium levels. The equilibrium is however now set by the new, lower set-point, indicating an important role of the kidneys in the very rapid regulation of the set-point for Ca^2+^ on bone surfaces.

**Figure 3:**
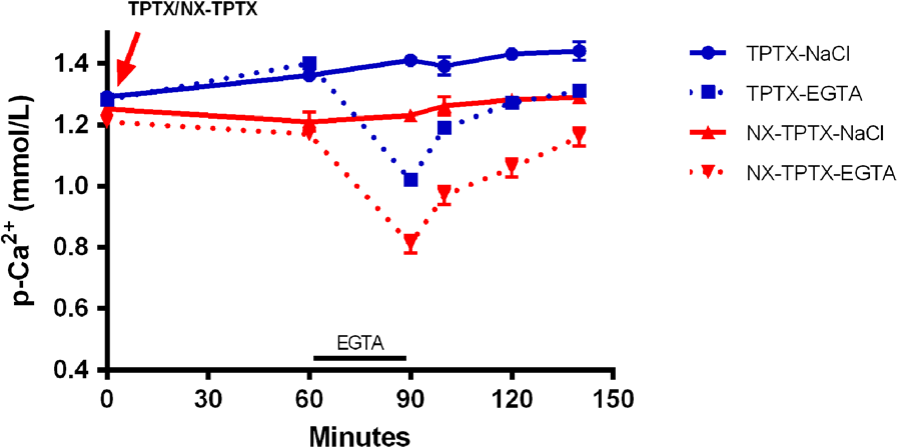
**Impact of the kidney on recovery of plasma Ca** ^**2+**^ **from acute hypocalcemia in thyroparathyroidectomized (TPTX) rats.** NX-TPTX rats had significantly lower nadir of EGTA induced hypocalcemia than that of EGTA infused TPTX rats (p < 0.01). Plasma Ca^2+^ concentration remained significantly lower in the NX-TPTX rats at all time points during recovery from hypocalcemia, (p < 0.01).

A possible direct or indirect effect of intact PTH, C-terminal PTH or CT was further examined by comparing the responses in TPTX and NX-TPTX rats, as shown in Figure 3. By performing TPTX the effect of changes in intact PTH, C-terminal PTH and CT is eliminated and in this way provides the possibility for a pure examination of the renal impact on the rapid plasma Ca^2+^ regulation. The nadir of hypocalcemia in the EGTA infused NX-TPTX rats was significantly lower than that of the EGTA infused TPTX rats, 0.81 ± 0.03 vs. 1.00 ± 0.02 mmol/L, (*p* < 0.01). Plasma Ca^2+^ concentrations were significantly lower in the NX-TPTX rats, as compared to the TPTX rats during the recovery from EGTA induced hypocalcemia, (*p* < 0.01) at time 100, 120 and 140 minutes. Thus, also in this experimental situation the NX group had a downward shift of the Ca recovery curve from acute hypocalcemia, once again providing further support for the existence of a rapid effect of NX on the set-point for Ca^2+^, which is independent of PTH and CT, although it still is respecting the degree of hypercalcemia, which developed post TPTX.

**Figure 4:**
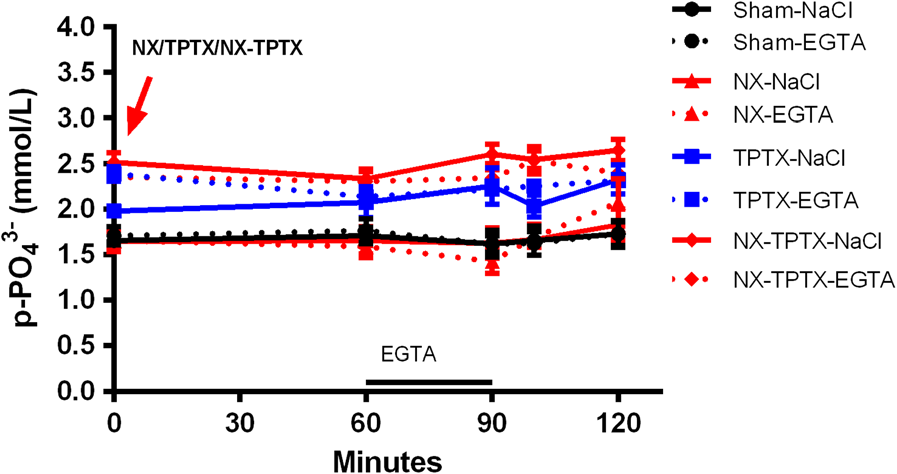
**Plasma-phosphate concentrations in NX, TPTX, NX-TPTX and sham rats.** Despite large deviations in plasma Ca^2+^ induced by NX, TPTX, NX-TPTX and EGTA infusion plasma P levels remained stable within the groups. The exception was in the NX-EGTA group, where a significant increase of plasma phosphate (p < 0.01) developed late in the experiment (please, see text).

The basal normal plasma P concentrations were more dispersed than basal Ca^2+^ values and differed from 1.74 ± 0.04 to 2.40 ± 0.6 mmol/L between the 8 groups of rats at time 0 min, i.e. before any experimental procedure was performed. After that plasma P remained stable within the groups for the next 120 minutes, except in the NX-EGTA group, where a significant increase of plasma P from 1.43 ± 0.14 to 2.08 ± 0.13 mmol/L (p < 0.01) took place, as indicated in Figure 4.

## Discussion

The results of present study clearly show that plasma Ca^2+^ falls rapidly very early after induction of acute uremia, and that plasma Ca^2+^ levels already 60 minutes after total nephrectomy became significantly decreased and further decreased for another 80 minutes. The mechanism for this rapid decline of plasma Ca^2+^ is not known. Hypocalcemia, as observed in patients with acute renal failure, was previously explained as being secondary to decreased synthesis of calcitriol and development of hyperphosphatemia [34,35]. The very early and rapid development of hypocalcemia, as observed in the present experimental model cannot, however, be explained as being due to changes in calcitriol or phosphate levels. Phosphate levels didn’t change and the half-life of calcitriol is too long, 6 hours. In the uremic condition the metabolic clearance rate of calcitriol is even lower, 57%, [36], even though these data were obtained in a human study on patients on chronic hemodialysis and not examined in acute uremia.

Our initial hypothesis was that this rapid development of hypocalcemia in a model of total nephrectomy was caused by accumulation of C-terminal PTH fragments and/or of CT.

Mammalian intact PTH is a polypeptide consisting of 84 amino acids [37-39]. The N-terminal region of PTH binds to the PTH1R [40], and is suggested to be the main part of the hormone with the major biological activity. Increasing evidence, however, indicate the existence also of a C-terminal PTH receptor (C-PTHR), that may have a significant biological effects, which are related to the Ca^2+^ homeostasis [40]. Circulating C-terminal PTH fragments are derived from the parathyroids and being a product of hepatic proteolysis of intact PTH as well [41,42]. The large C-PTH 7–84 fragment has been shown to have an effect opposite to intact PTH, lowering plasma Ca^2+^ [43,44]. The C-terminal PTH fragments are mainly cleared through the kidneys [45] as shown by our and other groups [46,47] and could theoretically accumulate in the acutely NX rats and thereby lead to the hypocalcemic response. Previously we have shown a rapid hypocalcemic effect of a large dose of PTH 7–84, which was seen already within one hour [33]. Similarly, could the metabolism of CT be diminished in NX rats, and thereby contribute to the aggravation of hypocalcemia [32]. An impact of C-terminal PTH fragments and/or CT on the rapid decline of plasma Ca^2+^ in acute uremia, as previously hypothesized from our lab, could however not be confirmed by the results of the present investigation, as no influence of PTH, C-terminal PTH and CT was found on plasma Ca^2+^ concentrations when comparing the models of TPTX (without PTH and CT) and NX-TPTX rats (without PTH, CT and kidneys). Thus, our present results interestingly points toward the existence of a kidney-bone axis, which is involved in the rapid regulation of plasma Ca^2+^, and that this very rapid regulation is not mediated by any of the classical calciotropic hormones, PTH and 1,25(OH)_2_D or CT.

Christov et al. recently added another interesting aspect to the possible existence of a rapid skeletal response to induction of acute uremia, [29], even though plasma Ca^2+^ levels were not followed in the study. In models of acute uremia they found an increase in FGF23 levels, which was observed already at one hour after acute kidney injury (AKI) [29]. This rapid elevation of FGF23 might further add to the existence of a kidney-bone axis, which is responding very rapidly to kidney injury. The P and Ca regulating hormone, FGF23, is mainly secreted from osteocytes. The osteocyte lineage, the lining cells, are covering all quiescent bone surfaces, where the labile exchangeable pool of calcium is supposed to take place [22]. As such results from the models of acute kidney injury [29] and the present investigation on NX rats point toward the existence of a rapid signal being exchanged between kidneys and osteocytes.

In the present study a decreased plasma Ca^2+^ level was observed in NX-TPTX rats compared to TPTX rats, indicating that the Ca^2+^ lowering effect of NX was independent of a PTH signal. Similarly, in the study of Christov et al. [29] elevation of FGF23 levels were found when AKI was induced in wild type mice, mice with osteocyte specific PTHR ablation or the deletion of PTH, indicating that the increase in FGF23 was not dependent on PTH signaling. Taken together, our results on NX rats and the results from AKI models suggest that PTH signaling is not necessary for the early skeletal response to acute uremia.

TPTX rats developed significant hypercalcemia, which is in line with our previous results [12]. This observation was originally made years ago by Copp, and lead to the discovery of CT [48]. The hypercalcemic response is caused by the eliminated CT tonus on bone, which apparently overrules the effect of PTX on bone [12]. The hypercalcemic effect of TPTX was diminished in the NX-TPTX group and NX-TPTX rats had lower plasma Ca^2+^ concentrations throughout the study, once again indicating that presence of kidneys influences the plasma Ca^2+^ concentration on a minute-to-minute basis.

EGTA is a specific calcium chelator that induces hypocalcemia by binding calcium in a stable physiological inert complex. The stability of the Ca^2+^-EGTA complex has previously been carefully examined by our group and it was clearly shown that the rapid recovery of plasma Ca^2+^ was not due to dissociation of calcium from the complex, not due to induction of a decreased plasma P and was not a product of alterations in plasma pH [10]. Thus EGTA binds Ca in blood with no subsequent dissociation of Ca. Whether the elimination of the renal clearance of EGTA in NX rats contributes to an accumulation of EGTA cannot completely be ruled out. Such an accumulation could theoretically have lead to an increased net amount of active EGTA and as such to an augmented Ca chelation and thereby to a more profound hypocalcemic response. In this respect it is important to notice that hypocalcemia also developed in NX rats that were not infused with EGTA. Furthermore, hypocalcemia in NX rats developed already at 60 minutes, which means before any intravenous infusion of EGTA.

Another question is whether plasma P levels are affected by the acute NX and thereby could have an effect on plasma Ca^2+^ regulation. Surprisingly, despite development of hypocalcemia NX rats had stable plasma P for 60 minutes post NX, clearly demonstrating that acute hypocalcemia was not balanced by acute recipocal hyperphosphatemia. Furthermore, plasma P remained stable independent of EGTA induced changes in plasma Ca^2+^ and independent of the presence of PTH. This dissociation between plasma Ca^2+^ and plasma P levels is in accordance with results on diurnal variations of the two ions [5].

Surprisingly, the basal normal plasma P concentrations were considerably more dispersed than the basal plasma Ca^2+^ values. At present this is not explained. Plasma P remained stable within the groups, except in the NX-EGTA group, where a significant increase of plasma P (p < 0.01) took place. Potentially this could be explained by a stimulatory effect of the EGTA induced hypocalcemia on the PTH secretion [49] in a situation where the increased PTH would result in release of P from the skeleton, but could not induce phosphaturia as the rats were NX.

The results of the current investigation clearly demonstrated that the kidneys affect the rapid changes in plasma Ca^2+^. The demonstration, that the induction of and recovery from hypocalcemia is significantly lower in NX rats, independently of PTH, C-terminal PTH and CT in the circulation, might indirectly indicate that presence of kidney is of significant importance for the setting the set-point for Ca^2+^ on bone surfaces. We, therefore, suggest that there might exist an as yet unknown factor or mechanism, which is involved in the very rapid signaling between kidney and bone, and which is affecting the minute-to-minute regulation of plasma Ca^2+^. We suggest that this factor acts rapidly as a hormone or a mechanism with a hormone-like effect on bone, and thereby sets the ‘set-point’ for plasma Ca^2+^ regulation on bone.

## Conclusion

In conclusion, our results clearly demonstrate that plasma Ca^2+^ concentrations on a minute-to-minute basis are influenced by the presence of kidneys. This indicates that the kidneys are of significant importance for the ‘set-point’ of plasma Ca^2+^ on bone independent of PTH and calcitonin. Our results point toward the existence of an as yet unknown factor/mechanism, which mediates signals between kidney and bone, and which is involved in the very rapid regulation of plasma Ca^2+^.

## Abbreviations

AKI: Acute kidney injury
Ca: Calcium
Ca^2+^: Calcium ion
Calbindin-D28K: Calcium-binding protein
Calcitriol: 1,25(OH)_2_D
CaR: Ca^2+^ sensing receptor
C-PTHR: C-terminal PTH receptor
CT: Calcitonin
C-terminal PTH: Carboxyterminal fragment of PTH
ECF: Extracellular fluid
EGTA: Ethylene-bis(oxyethylenenitrilo)tetraacetic acid
FGF23: Fibroblast growth factor 23
N-terminal PTH: Amino terminal fragment of PTH
NX: Bilateral nephrectomy
NX-TPTX: Bilateral nephrectomy and thyroparathyroidectomized
P: Phosphate
PTH: Parathyroid hormone
PTH1R: PTH/PTHrP receptor (PTH/parathyroid hormon related peptide receptor)
PTX: Parathyroidectomized
TPTX: Thyroparathyroidectomized
TRPV5: Transient receptor potential cation channel subfamily V member 5
TX: Thyroidectomized
VDR: Vitamin D receptor
